# Development of synthetic biotics as treatment for human diseases

**DOI:** 10.1093/synbio/ysac001

**Published:** 2022-01-31

**Authors:** Aoife M Brennan

**Affiliations:** Synlogic Operating Company, Cambridge, MA, USA

**Keywords:** synthetic biology, therapeutics, synthetic biotic medicines

## Abstract

Advances in synthetic biology have allowed the generation of strains of bacteria that are genetically altered to have specific therapeutic benefits. These synthetic biotics, also widely referred to as engineered living therapeutics, have tremendous potential as a new therapeutic modality, and several have advanced into the clinic and human testing. This review outlines some of the unique attributes of synthetic biotics as well as some of the challenges in their development as prescription products. Regulatory considerations are discussed, and a case study of a program that has advanced into Phase 2 testing is provided: SYNB1618 for the treatment of PKU.

## 1. Introduction

Despite recent advances in the understanding of the biological basis of disease, many people suffer from conditions with no or inadequate treatment. As a mechanism to address this unmet medical need, the role of the bacteria that colonize the human body (the human microbiota) has recently gained attention. Host–microbe interactions have been implicated in many diseases—most notably metabolic diseases, inflammatory diseases and cancer (1, 2). Many approaches are being pursued to advance therapies based on this science, including identification of consortia of naturally occurring bacteria (3), identification of single strains of naturally occurring bacteria (4), development of phages that deplete specific disease-causing members of the microbiota (5) and development of small molecules that modify the behavior of bacteria within the microbiota (6).

An alternative to searching for naturally occurring bacteria that ameliorate a disease is to use the tools of synthetic biology to engineer bacteria with specific functionality based on the understanding of disease biology. These engineered bacteria are called synthetic biotics. In this review, I will discuss the opportunities and challenges of developing synthetic biotics as well as some unique regulatory considerations.

## 2. Synthetic biotics

A synthetic biotic is a bacterium that has been genetically altered to perform a specific function for diagnostic or therapeutic benefit. Advantages of this approach compared to leveraging existing members of the microbiota include the ability to select a chassis with good manufacturing feasibility as well as a history of safe use in humans; the potential to precisely engineer mechanisms of action and specific potency; the use of disease-relevant promoters that regulate the effector function in the appropriate context (i.e. genetic circuit) and the use of auxotrophies or other genetic modules to control growth *in vivo* and in the environment (7, 8).

The design process starts with understanding the specific disease that the synthetic biotic is intended to treat and the development of a pharmacological hypothesis for how the pathophysiology will be intercepted at the specific site of desired action. Several classes of effectors have been engineered into bacteria, including enzyme pathways that metabolize disease-causing toxins (9), production of protein effectors (10), and production of small-molecule effectors (11). Synthetic biotics have been engineered for application to the skin (12) and for intra-tumoral injection in oncology (11, 13), but the area of most active research is the development of synthetic biotics designed to work from the gut lumen (10, 14), as shown in Table 1. Due to the active cross-talk between the gastrointestinal (GI) tract, metabolic pathways, the nervous system and the immune system, oral synthetic biotic medicines can be designed to treat GI tract–specific as well as systemic diseases.

**Table 1.** Examples of synthetic biotics that have entered clinical development

Synthetic biotic	Chassis organism	Indication	Route of administration	Sponsor organization	Country of origin
AG013	*Lactococcus lactis*	Oral mucositis	Oral mouth wash	Oragenics	USA
AG019	*Lactococcus lactis*	Type 1 diabetes	Oral	ActoBio Therapeutics	Belgium
ADXS-HOT	*Lysteria monocytogenes*	Non-small cell lung cancer	Intravenous	Advaxis Immunotherapies	USA
AZT-04	*Staphylococcus epidermidis*	Cancer associated rashes	Topical	Azitra	USA
SYNB1618	*E. coli* Nissle 1917	Phenylketonuria (PKU)	Oral	Synlogic	USA
SYNB8802	*E. coli* Nissle 1917	Enteric hyperoxaluria	Oral	Synlogic	USA
SYNB1891	*E. coli* Nissle 1917	Solid tumors and lymphoma	Intra-tumoral	Synlogic	USA
NOV-001	*Bacteroides*	Enteric hyperoxaluria	Oral	Novome	USA

## 3. Synthetic biology considerations in the development of synthetic biotics

**Figure 1.**
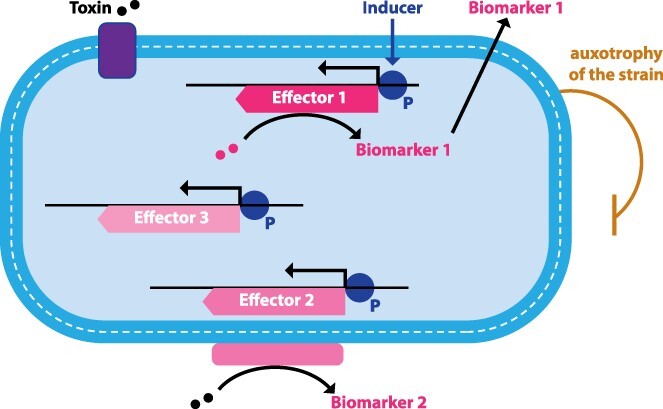
Components of a synthetic biotic.

As shown in Figure 1, a synthetic biotic is composed of a chassis organism, one or more genetic circuits required for therapeutic effect (i.e. effectors), and other ancillary elements such as transporters and auxotrophies.

One of the most important considerations for the development of synthetic biotics is the choice of chassis organism into which genetic circuits will be engineered. Unsurprisingly, laboratory-domesticated *Escherichia coli* strains such as K-12 have been used for the development of many *in vitro* synthetic biological systems. However, commensal bacteria may make a superior choice for human *in vivo* applications because they have co-evolved with humans, and strains already in use as probiotics are familiar to consumers and regulatory agencies such as the Food and Drug Administration (FDA) and the European Medicines Agency (EMA) (15, 16). The environment in which the biotic is expected to function (skin, GI tract, tumor, etc.), the ease of its genetic manipulation and its ability to be manufactured at scale are also crucial factors that must be appropriately balanced.

A core component is the genetic circuit, which is composed of DNA sequences that encode biological parts designed to perform logic functions akin to a computer program executing an algorithm (8, 17, 18). In simple terms, a genetic circuit receives and converts an input signal into a quantifiable output. In the case of a synthetic biotic, these outputs may be utilized to perform an array of useful biological functions that are relevant to the treatment of disease (19). The circuit’s mechanistic activity can be referred to as its ‘effector function’. The input, or ‘switch’ as it is often called, is typically controlled by an inducer–promoter pair. Bacterial promoters display species specificity, so the availability of such tools can be a key consideration in the selection of a chassis organism.

The switch composition of a genetic circuit is largely dependent on the level of effector activity required and the environment in which it is expected to function. While industrial applications of engineered bacteria may use small-molecule inducers such as isopropyl β-D-1-thiogalactopyranoside (commonly referred to as IPTG), these small-molecule inducers may not be optimal for synthetic biotics that are expected to function in specific locations within the human body. For this reason, *in vivo* switches that are capable of sensing and responding to signals encountered within the human body have been developed. These can sense physiological states—for example, pH, temperature or oxygen tension (8, 15, 19). Disease-specific switches have also been constructed, such as those sensing inflammation-dependent signals like reactive oxygen species, tetrathionate or nitrate (15, 20, 21).

Other synthetic switches have been developed that permit self-sensing by engineered bacteria through repurposing of bacterial quorum sensing systems (15, 17). Quorum sensing systems typically depend on the production of small molecules or peptides called ‘autoinducers’ that accumulate in the environment as the cell population increases. The autoinducer modulates gene expression when the bacterial population reaches a critical density. In this manner, bacteria can be programmed to switch on their effector function only when an effective cell concentration has been established (15). This may be useful in synthetic biotics whose effector function is toxic or could cause unwanted side effects, ensuring that the engineered activity is delivered only to disease-specific environments that support growth and division (22, 23).

An additional consideration when designing a synthetic biotic is the need for a specific strategy around biocontainment. This can be important from an environmental perspective, but there can also be advantages to limiting growth in human recipients of synthetic biotics (7). A number of strategies have been developed, including the following:

- Engineering nutritional auxotrophy involving deletion of an essential chromosomal gene in the chassis to make it dependent on an exogenous supply of an essential metabolite (16, 24).
- Synthetic amino acid auxotrophy that recodes strains, making them dependent on an exogenously supplied synthetic amino acid (24).
- Synthetic nucleic acid auxotrophy where, for example, a strain is evolved to be dependent on chlorouracil (25).
- Kill switches that use logic-based genetic circuits programmed to sense exogenous inputs to determine if the requirements for cell proliferation continue to be met (26).

To date, more sophisticated biocontainment strategies have not been deployed in therapeutic applications because they generally have escape rates higher than the 10^−8^ frequency recommended by the National Institutes of Health (15, 16). For this reason, simple nutritional auxotrophy has remained the biocontainment strategy of choice for clinical-stage programs.

Finally, it is important to consider genetic stability when constructing a strain that will ultimately be administered to humans. Plasmids are excellent tools for quickly manipulating and interrogating genes of interest; however, plasmid DNA is not particularly stable and will typically be lost in the absence of antibiotic selection. The FDA also discourages the use of antibiotic markers in live biotherapeutic products (LBPs) (27), further discouraging the use of plasmids to carry engineered genetic circuits. In addition, plasmids are prone to unequal segregation during rounds of cell division (15). For these reasons, integration of engineered circuits directly into the chassis chromosome may be a preferred approach.

## 4. Challenges in the development of synthetic biotics as drugs

The tools of synthetic biology have advanced tremendously in the past decade, with the reduction in the cost of sequencing and printing DNA and the development of libraries of reusable parts for multiple chassis organisms (28, 29). Lagging is our understanding of the specific mechanisms driving the relationship between the bacteria that live on and in us and disease. Identification of new bacterial target effectors and their validation as truly disease modifying is the next challenge for microbiome research (2). This challenge is further amplified by the frequent lack of translatability of microbial approaches between animal models and humans (30). Ultimate validation will only be delivered by human interventional studies, but a combination of multiple human observational data sets (genetics, transcriptomics, metabolomics, etc.) aided by the development of more translationally dependable preclinical models including organ-on-chip systems can help minimize the risk of some of these new therapeutic targets (1, 30, 31). Once these novel targets are identified, synthetic biology can be used to rapidly generate clinical strains ready for development as drugs.

Manufacturability is another key consideration in the development of synthetic biotics and needs to be incorporated into every decision from the selection of the chassis organism all the way through to approval. It is currently difficult to predict whether specific genetic changes will induce a growth defect requiring frequent iteration during the design stage. Similarly, promoters that may work well in flasks and at small scales may not perform well at the large scales required to meet clinical demand for eventual commercialization (32). The dose of cells required will depend on the potency of the specific strain, i.e. how well it performs its effector function. Balancing potency, dose and manufacturing feasibility requires collaboration across different team members. As we generate more data on genotype–phenotype and manufacturability, curation of these data sets so that they can be a useful source of future predictive or artificial intelligence-based algorithms that can inform design choices could be hugely valuable.

Like all drug development platforms and programs, it is critical to keep the eventual patient in clear view throughout development. Spending time to understand patient needs at the outset of a project and checking in frequently throughout development to ensure those needs are being considered is important to success (33). Factors to be considered include the following:

1. Understanding of endpoints that are meaningful to patients in deciding whether to use a new therapy. These may or may not be the same endpoints that are important to prescribers and regulators.
2. Formulation and delivery for example frequency of administration.
3. Understanding perceptions of the technology and how to explain it so that it is accessible to the patient community.

The importance of open engagement with the patient community is particularly important, given the potential for negative perceptions related to use of genetically modified organisms. Thoughtful design decisions, transparency, and clear and frequent communication with the patient community are ways to mitigate this risk (34).

## 5. Regulatory considerations

Delivering on the therapeutic potential of synthetic biotics requires a transition from research to studies in humans. A key gate to the initiation of human clinical trials is the approval of a national competent authority and ethics committees in the jurisdiction where the trial will be conducted.

Bacterial therapeutics are classified as LBPs, which are defined as live organisms developed to treat, cure or prevent a disease or condition in humans. Notably, LBPs exclude vaccines, filterable viruses, oncolytic viruses and organisms used as vectors for transferring genes into the host (35). Genetically modified bacteria are classified as recombinant LBPs. In the United States, recombinant LBPs are regulated by the FDA through the Center for Biologics Evaluation and Research. In 2016, the FDA issued a guidance document describing the regulatory considerations for conducting clinical trials with LBPs (36). The European Pharmacopeia published a monograph setting the quality standards for LBPs for human use, in European Pharmacopoeia, Supplement 9.7, effective in April 2019 (37). While no synthetic biotic has been approved for commercial sale, several have been approved by global competent authorities for clinical evaluation as outlined in Table 1.

The first step in the clinical evaluation of any potential therapeutic product is the clinical trial application or investigational new drug (IND) application (38). Consultation prior to submission of the application to initiate clinical trials and regularly throughout development is critical in this new area, as there is minimal precedent, current regulatory guidance includes general considerations only and there is lack of consensus on requirements across regions and states. Each LBP will have unique properties, including route of administration, pathogenicity potential, colonization, clearance and microbial products. These factors and others will influence the data required to support the benefit–risk evaluation by regulators (35). For example, bacterial components such as lipopolysaccharides will result in different risks for an oral product compared to a product that is intended to be administered intravenously or intratumorally. In addition, while some effector functions such as those generating microbial products that are generally recognized as safe (39) may not require preclinical toxicological evaluation, other effector functions may require testing prior to the initiation of human trials.

One of the important safety risks to consider when assembling the clinical trial application or IND is the potential for infection with the engineered organism. The risks for infection can be influenced by the biodistribution of the product as well as the intended patient population (35). Preclinical distribution studies following administration that is representative of the intended clinical route may be required to support a clinical trial. Demonstrating sensitivity to commonly prescribed antibiotics is also important and can guide treatment of any infection or possible infection in the clinic (40).

In common with all investigational medicinal products, the manufacturing facility where the engineered microbe is manufactured should operate under Good Manufacturing Practices (41). While the framework can be shared with other biologics, there are a number of unique manufacturing considerations when preparing to conduct a clinical trial with a synthetic biotic, including the following:

- The genetic sequence of exogenously introduced genes must be provided in regulatory filings, including a high-quality, complete genome sequence for the engineered clinical candidate strain.
- Evidence supporting the stability of strain modifications over time, particularly during fermentation will be required for regulatory filings.
- The ability of the organism to replicate or persist in the environment, as well as any biocontainment strategies incorporated into the engineering may influence the regulatory path and the data required to support initiation of clinical trials.
- Assays to determine the absence of contamination by adventitious agents are required for all products but may be particularly challenging for a living bacterial–based product. The required sensitivity (particularly for products intended for non-oral routes of administration) usually requires culture-based methodology.

Following completion of the IND process in the USA, regional ethics committees or institutional review boards will review the clinical protocol and other study documents. Because engineered bacteria are considered biological agents, review by institutional biosafety committees is frequently required (42). The purpose of this committee is to assess the environmental risk of the product and the ability of the engineered bacteria to survive and replicate in the environment as well as any potential toxicity should an unintended person be exposed (7).

This environmental review differs globally, so it is important to understand the process in the region where the clinical trial will take place. The lack of a standardized set of requirements and procedures can place a high administrative burden on investigators and sponsors intending to conduct a trial across multiple international sites. In the European Union, while there is consensus regarding the conduct of clinical trials and a central procedure for marketing authorization, the interpretation of the various directives regarding genetically modified organisms used in clinical research is variable, with lack of consensus for what constitutes contained use versus deliberate release. A program that is determined to constitute deliberate release in a given state will require a more extensive review and approval process (43, 44).

As with other therapeutic products, approval and licensure require the demonstration of safety, efficacy (from well-controlled clinical trials) and quality (i.e. reliability, robustness and consistency of each batch produced). While there is currently a paucity of regulatory precedent in this area, we can learn from recent experience with other complex products like gene and cell therapy, where delays in the peri-approval period have been due to manufacturing issues and difficulty demonstrating comparability between the product used in clinical trials and the product intended for commercialization (45). Early investment in a robust set of assays and in process development as well as regular interactions with the regulatory agencies will likely be key to success.

## 6. Development of a synthetic biotic for the treatment of PKU: a case study

Phenylketonuria (PKU) is an inborn error of metabolism caused by a genetic defect in the gene encoding phenylalanine (Phe) hydroxylase, an enzyme responsible for converting Phe to tyrosine. If not detected early and treated by a strict Phe-controlled diet, PKU can result in irreversible neurological damage (46). Phe is a component of most dietary protein and is also known to circulate back into the gut from systemic circulation, making it potentially treatable by an orally administered, gut-restricted synthetic biotic that degrades Phe (47).

**Figure 2.**
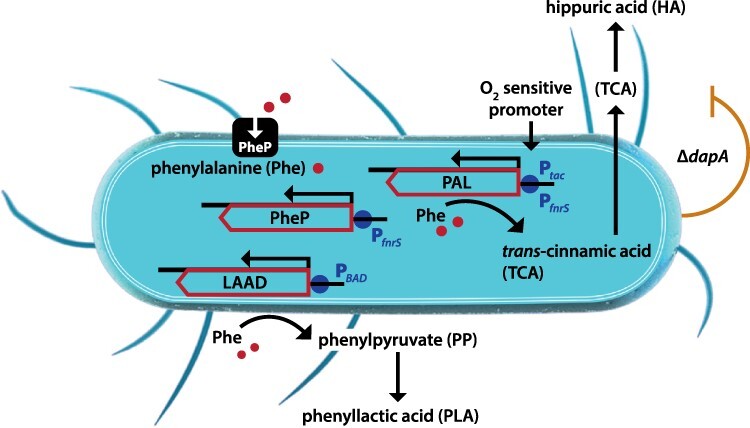
Two-pathway metabolism of phenylalanine by SYNB1618, an engineered *Escherichia coli* Nissle.

To create a synthetic biotic suitable for the treatment of PKU, the strain engineering team at Synlogic constructed SYNB1618, a Phe-degrading derivative of *E. coli* Nissle 1917 (EcN). Two pathways for Phe degradation were engineered into EcN (Figure 2). The first pathway uses phenylalanine ammonia lyase (PAL) to convert Phe to *trans*-cinnamate (TCA), a harmless metabolite. PAL is a cytosolic protein, requiring Phe transport into the cell; therefore, expression of a high-affinity Phe transporter, PheP, was added to increase the intracellular availability of Phe. The second Phe degradation pathway that was engineered uses l-amino acid deaminase, a membrane-associated enzyme that converts Phe to phenylpyruvate. The genes encoding these enzymes and transporter were integrated into the EcN chromosome under the control of an inducible promoter to enable the control of gene expression and maintenance of genetic integrity during the high-density, large-scale growth needed to prepare doses for humans. Finally, SYNB1618 contains a deletion of the *dapA* gene, encoding 4-hydroxytetrahydropicolinate synthase, which enables biocontainment by rendering engineered bacteria dependent on exogenous diaminopimelate for cell wall biosynthesis and growth (9). SYNB1618 was not our first or even our second attempt to develop a Phe-consuming strain. A prior version had gone through extensive testing but fell at the final fence when the external manufacturing vendor would not accept the strain into their facility due to failure on a phage test. Some genetic detective work identified the prophage that was successfully deleted from the strain that became SYNB1618.

SYNB1618 underwent extensive preclinical testing prior to initiation of clinical trials, demonstrating reduction of blood Phe concentrations in the Pah^enu2/enu2^ PKU mouse model of PKU and a reduction in the postprandial increase of blood Phe in healthy cynomolgus monkeys after an oral Phe challenge (9, 48). As we developed our preclinical package, we requested guidance from the FDA in the form of a pre-IND meeting, which was very valuable in understanding the specific requirements for initiation of a clinical trial.

These discussions informed the toxicology studies and extensive genetic and *in*  *vitro* characterization of the strain that supported the submission of an IND application to the FDA, allowing SYNB1618 to be evaluated in humans.

The initial clinical trial was a Phase 1/2a randomized, placebo-controlled study in adult healthy volunteers and patients with uncontrolled PKU using a frozen liquid formulation of SYNB1618. The primary outcome of the study was safety and tolerability, and the secondary outcome microbial kinetics. A D5-Phe tracer was used to study exploratory pharmacodynamic effects. SYNB1618 was safe and well tolerated; mild-to-moderate GI adverse events were observed at higher doses. SYNB1618 rapidly cleared from the GI tract after discontinuation of dosing; no fecal sample was above the limit of quantification 4 days after the last dose. Consistent with the preclinical data, we observed dose-responsive increases in strain-specific Phe metabolites in plasma (TCA) and urine (hippuric acid), providing proof-of-mechanism that the strain was active in the GI tract and consuming Phe (49).

The next step in the development of SYNB1618 as a potential therapeutic for patients with PKU was to develop a formulation that would be stable at room temperature or domestic refrigeration, recognizing that the product would need to be taken multiple times daily to control Phe levels in patients. Synthetic biotics are complex living cells, and we needed to develop assays to evaluate the impact on the activity and health of the cells prior to initiating process development work. Following evaluation of a number of methods to preserve the cells without requiring −80°C refrigeration, we focused on lyophilization, which freeze dries the cells to an off-white powder that can then be further filled into sachets, capsules or pressed pills. We developed a lyophilization process that preserved cell activity and viability and demonstrated good stability at 2–8°C and at room temperature (48). This development supported the initiation of additional clinical work that could include out-patient studies of longer duration in patients with PKU. These studies were planned with the active involvement of the patient advocacy community who provided valuable input—for example, the advice that patients with PKU prefer sachets to pills.

While SYNB1618 continues to be evaluated in the clinic in an ongoing trial in patients with PKU, it also provides a rich data set for the development of predictive models that can be applied to other synthetic biotics in development. As noted above, one of the challenges of the development of synthetic biotics is the ability to predict activity within the dynamic environment of the human GI tract. Species-specific differences in GI physiology, as well as limitations in the ability to continuously sample the site of action mean that, while animal models can be useful, they cannot fully address all relevant translational questions. Our team has used the SYNB1618 and other Phe-consuming prototypes to develop *in*  *vitro* simulation models like gut-on-chip (33), as well as predictive pharmacology models to predict the Phe-consuming activity of the strain *in*  *vivo* in humans (50). With additional data from patients with PKU as well as inputs from strains developed with other effectors, these model systems can continue to increase in utility and predictive power.

## 7. Opportunities and future directions

Synthetic biology as a discipline will have a tremendous impact on global health across diagnostics, vaccines and therapeutics over the coming decades (52). From enabling discovery to optimizing production in cell-based systems, the proportion of therapeutics where synthetic biology has contributed could grow exponentially. Synthetic biotics represent the application of synthetic biology tools to bacterial therapeutics, which holds promise for treating disease in new ways, offering first treatments or better treatments to patients.

Synthetic biotics are living machines that can be engineered for potency and to respond to local conditions at the site of action in the human body based on either physiological or pathological signals. This can enable delivery of a therapeutic in a targeted way, delivering the right effector at the right place at the right time. These closed-loop systems will require continued development of disease-specific sensors and matching effectors, but they hold tremendous promise.

An important challenge for many chronic and complex diseases is that multiple biological pathways may play a role, requiring combinations of treatments. These combinations often come with a high cost in terms of health-care dollars, toxicity risk and patient adherence to polypharmacy. Synthetic biotics could be designed to produce multiple effectors in a single product under the control of separate sensors if needed. As our understanding of the underlying pathophysiology of these diseases improves, synthetic biotics could offer a unique opportunity to simultaneously address multiple disease processes.

Another key advantage of using a therapeutic self-replicating, cellular system is the ability to greatly reduce patient burden and increase compliance by engineering synthetic biotics that can stably colonize a microbial niche. This could enable a one-time application that could be particularly beneficial for chronic medical conditions or for preventative health applications. Understanding the rules of colonization as well as how to maintain genetic stability over the long term will be critical in achieving this goal, particularly as many chassis organisms currently used are not good colonizers and effector functions can be costly metabolically, potentially creating negative selection pressure (51).

We are entering an incredibly exciting time for synthetic biotics with multiple programs and approaches in the clinic. Advances in synthetic biology will continue to drive greater efficiency in discovery and development as well as breadth in the range of diseases that can be addressed.
